# Real-world treatment patterns and clinical outcomes in patients with radioiodine-refractory differentiated thyroid cancer (RAI-R DTC) treated with first line lenvatinib monotherapy in the United States

**DOI:** 10.1007/s12020-023-03638-7

**Published:** 2023-12-16

**Authors:** Francis Worden, Olivera Rajkovic-Hooley, Neil Reynolds, Gary Milligan, Jingchuan Zhang

**Affiliations:** 1 University of Michigan Health System, Ann Arbor, MI USA; 2 Adelphi Real World, Bollington, UK; 3 Eisai Inc., Nutley, NJ USA

**Keywords:** Differentiated thyroid cancer, Radioiodine-refractory, Lenvatinib, Real-world effectiveness, Chart review, United States

## Abstract

**Purpose:**

Lenvatinib was approved for the treatment of patients with radioiodine-refractory differentiated thyroid cancer (RAI-R DTC) in the United States (US) in 2015. The main objective of the current study was to assess real-world clinical effectiveness in RAI-R DTC patients treated with first line lenvatinib monotherapy in the US.

**Methods:**

A retrospective chart review was conducted in RAI-R DTC patients who initiated lenvatinib monotherapy as first line treatment between February 2015 and September 2020. Anonymized data were abstracted by prescribing physicians from individual patient’s electronic health records. Clinical outcomes included provider-reported real-world best overall response (rwBOR), real-world progression-free survival (rwPFS), and overall survival (OS). Time-to-event endpoints were assessed using Kaplan–Meier methods.

**Results:**

Our study included 308 RAI-R DTC patients treated with first line lenvatinib. At lenvatinib initiation, patients’ median age was 60 years, 51.6% were female, and 26.0% of patients had an ECOG performance score of ≥2. Over the follow-up period, 32.5% of patients discontinued first line lenvatinib permanently, with others remaining on treatment. The median duration of lenvatinib therapy was 17.5 months overall. Provider-reported rwBOR (complete or partial response) to lenvatinib was 72.4%. Median rwPFS was 49.0 months. Estimated rwPFS rates at 24 and 48 months were 68.5% and 55.0%, respectively. Estimated OS rates at 24 and 72 months were 78.4% and 57.0%, respectively; median OS was not reached.

**Conclusion:**

The current study reinforces the clinical effectiveness of first line lenvatinib as standard of care in patients with RAI-R DTC in real-world clinical practice in the US.

## Introduction

Thyroid cancer is the eighth most common cancer globally, with more than 580,000 new worldwide cases diagnosed, and more than 43,000 deaths reported in 2020; of these, 62,000 of new cases and 2,400 deaths were in the United States (US) [1, 2]. Differentiated thyroid cancer (DTC), comprising follicular, papillary and Hürthle cell (oncocytic) thyroid cancer, is the most prevalent and accounts for more than 90% of all thyroid cancer cases [3]. The current treatment for DTC consists of surgery and radiotherapy, including radioactive iodine^131^ (RAI). However, approximately 5–15% of DTC, and 50% of metastatic DTCs, are non-responsive or refractory to RAI therapy (RAI-R). RAI-R DTC patients are identified as those in whom no RAI uptake is present at the malignant/metastatic tissue on a diagnostic scan or from images several days after RAI treatment, or the tumor tissue loses the ability to concentrate RAI after previous evidence of RAI-avid disease, or RAI is only present in some, but not other, tumor foci, or DTC metastases progress despite a significant concentration of RAI [4].

In the setting of symptomatic disease or progression not amenable to RAI therapy, the NCCN Guidelines® recommend systemic therapy [5]. Once DTC becomes refractory to RAI treatment, patients’ overall prognosis is poor, with 10-year survival rates of less than 15% [6]. Systemic treatments for thyroid cancers have been evolving over the last decade, driven by the increased understanding of the underlying molecular mechanisms of these tumors, specifically in DTC [7]. Lenvatinib, a multi-targeted tyrosine kinase inhibitor, was approved in the United States (US) in 2015 as monotherapy for the treatment of patients with progressive, locally advanced, or metastatic DTC that is refractory to RAI treatment [8].

Approval was based on efficacy results from the randomized phase III SELECT clinical trial, in which patients who received lenvatinib had a better objective response rate (ORR) compared to those in the placebo group (64.8% vs 1.5%, respectively, *p* < 0.001), and significantly longer progression free survival (PFS, 18.3 months vs 3.6 months, respectively) [9]. In a subset analysis, an overall survival (OS) benefit was subsequently observed with the use of lenvatinib (HR = 0.53 versus placebo; 95% CI: 0.31–0.91; *p* = 0.02) in patients with RAI-R DTC older than 65 years of age [10]. In a later retrospective analysis of SELECT, patients with a lower tumor burden at baseline who received lenvatinib had prolonged OS versus those with a higher tumor burden (median OS not reached vs 29.1 months, respectively; HR, 0.42; 95% CI, 0.28–0.63) [11].

Lenvatinib is currently recommended in the 2022 NCCN Guidelines® as the preferred, category 1 treatment option for treatment of RAI-R DTC [5]. Real-world data documenting treatment patterns and clinical outcomes in patients with RAI-R DTC who were treated with lenvatinib monotherapy, however, are limited. In recent years, several new systemic therapies have been approved by the FDA for the treatment of advanced RAI-R DTC patients, in particular those whose tumors harbor specific genetic alterations, such as RET fusion, NTRK fusion, and BRAF mutation. Since treatment paradigms continue to evolve, we conducted this study to assess the real-world treatment patterns and related clinical characteristics and clinical outcomes in patients with RAI-R DTC in the US treated with first line (1L) lenvatinib monotherapy.

## Methods

### Study design and patient population

Our study was a retrospective chart review of patients with RAI-R DTC in the US who had initiated lenvatinib monotherapy as first line treatment in routine clinical practice. A geographically representative sample of oncologists was recruited to participate, provided they were personally responsible for treatment decisions and management of patients with RAI-R DTC and had qualifying patients. Patient inclusion criteria for eligibility included a histologically confirmed diagnosis of DTC, a clear physician-reported diagnosis of RAI-R status before initiating lenvatinib treatment, aged ≥18 years at the initiation of lenvatinib monotherapy, lenvatinib monotherapy for RAI-R DTC initiated in 1L between February 13, 2015 and September 30, 2020, and a complete treatment history available from initiation of lenvatinib to last follow-up. Patients were excluded if they had received lenvatinib for RAI-R DTC as part of a clinical trial, if they received any systemic treatments for primary tumors other than DTC during the study period, or if they had synchronous anaplastic histology at diagnosis.

Data were extracted by participating physicians from individual patients’ electronic health records and captured via a secure electronic online case report form. In order to avoid selection bias, physicians were instructed to randomly select five patients or fewer from those meeting the inclusion criteria (both alive and deceased patients). Qualifying patients were then chosen as those whose first name initial matched a computer-generated random letter. Physicians with more than one eligible patient had to place those patients in alphabetical order based on their first name.

### Study variables and statistical analysis

The baseline patient demographics and clinical characteristics, treatment patterns, and real-world clinical outcomes were evaluated. Clinical outcomes on lenvatinib treatment included real-world treatment response, real-world progression-free survival (rwPFS), and OS. Real-world treatment response and progression were collected directly from the patient’s medical record as reported by the treating physician at the time of assessment. Details of specific criteria used by the treating physician for the assessment of response and progression (e.g., RECIST, radiology scans, and/or other patient or clinical factors) were also collected. Real-world treatment response was recorded as complete response (CR), partial response (PR), stable disease (SD), or progressive disease (PD) [12].

Real-world overall response rate (rwORR, comprising CR and PR) and real-world disease control rate (rwDCR, comprising CR, PR, and SD) were calculated. rwPFS was defined as the time from the initiation of lenvatinib treatment to the earliest date of progression or death whilst on treatment; patients without a progression or death event were censored at the start of the next treatment line or last follow-up. OS was defined as the time from initiation of lenvatinib treatment until the date of death for any reason; patients without a death event were censored at last follow-up.

Descriptive statistics were reported with categorical variables presented as frequency and percentage and continuous variables presented as means and standard deviations (SD) and/or medians with interquartile ranges (IQR) or 95% confidence intervals (CI). Kaplan–Meier analyses were conducted to analyze time-to-event outcomes including rwPFS and OS. All analyses were conducted in Stata Statistical Software 17 0 5 (StataCorp. 2021. College Station, TX: StataCorp LLC).

## Results

### Demographics and clinical characteristics

A total of 65 physicians provided all patient data for the study, including 81.5% medical oncologists and 18.5% oncologists specializing in endocrine diseases or Ear, Nose and Throat. Over 85% of physicians had been in practice for more than 10 years, with 67.7% in urban, 29.2% in suburban, and 3.1% in rural practices. Physicians were recruited from different practice types (academic 38.5%, community hospital 32.3%, community private practice 29.2%), and across all geographic regions of the US (Northeast 29.2%, Midwest 18.5%, West 20.0%, Southeast 16.9%, Southwest 15.4%). The mean (SD) physician caseload was 55 (66) RAI-R DTC patients.

Data for 308 patients with RAI-R DTC treated with 1L lenvatinib were included for analyses. Demographics and clinical characteristics for patients are shown in Table 1. Overall, 48% of patients were male, with a median age of 60 years at 1L lenvatinib initiation. Approximately three quarters (73.4%) of patients were Caucasian, 15.6% African American, and 4.9% Asian; 16.2% were Hispanic/Latino. At the time of lenvatinib initiation for RAI-R DTC, 72% of patients had an Eastern Cooperative Oncology Group (ECOG) score of 0–1 and 26% had a score of ≥2. Metastases were mainly reported in the lymph nodes (42.9%) and lung (33.1%). Hypertension and diabetes were reported in 34.4% and 12.3% of patients, respectively.

**Table 1:** Demographics and clinical characteristics at 1L lenvatinib initiation for patients with RAI-R DTC

	Overall (*N* = 308)
Age at lenvatinib initiation^a^, median age	60
Gender, *n* (%)	
Male	149 (48.4)
Female	159 (51.6)
Race, *n* (%)	
White/Caucasian	226 (73.4)
Black/African American	48 (15.6)
Asian	15 (4.9)
Other	19 (6.1)
Ethnicity, *n* (%)	
Hispanic/Latino	50 (16.2)
Not Hispanic/Latino	258 (83.8)
Locally documented histology, *n* (%)	
Follicular thyroid cancer	149 (48.4)
Papillary thyroid cancer	149 (48.4)
Hürthle cell thyroid cancer	10 (3.2)
ECOG performance status score at initiation if 1L lenvatinib	
0	76 (24.7)
1	147 (47.7)
2	64 (20.8)
3	14 (4.5)
4	2 (0.6)
Unknown	5 (1.6)
AJCC disease stage^b^, *n* (%)	
<55 years: Stage I (any T, any N, M0)	21 (6.8)
Stage II (any T, any N, M1)	72 (23.4)
≥55 years: Stage I (T1/T2 N0/NX, M0)	20 (6.5)
Stage II (T1-T2, N1, M0 or T3a/T3b, any N, M0)	53 (17.2
Stage III (T4a, any N, M0)	29 (9.4)
Stage IVA (T4b, any N, M0)	25 (8.1)
Stage IVB (any T, any N, M1)	84 (27.3)
Unknown	4 (1.3)
Site of metastases, *n* (%)	
Lymph nodes	132 (42.9)
Lung	102 (33.1)
Bone	62 (20.1)
Liver	47 (15.3)
Brain	13 (4.2)
Breast	9 (2.9)
Skin	4 (1.3)
Vital status at end of follow-up, *n* (%)	
Alive	231 (75.0)
Deceased	71 (23.1)
Unknown	6 (1.9)

*1L* first line, *AJCC* American Joint Committee on Cancer, *DTC* differentiated thyroid cancer, *ECOG* Eastern Cooperative Oncology Group, *RAI-R DTC* radioiodine-refractory differentiated thyroid cancer

^a^Excluding patients >90 years of age

^b^AJCC disease staging was referenced. https://www.ncbi.nlm.nih.gov/pmc/articles/PMC5467103/

Approximately one third of patients (35.4%) had recorded surgery prior to RAI-R diagnosis, predominantly total thyroidectomy (93.6%) and neck lymph node removal (54.1%). All patients had received RAI therapy, with a large majority of patients (85.6%) having previously received a single treatment and a small minority (14.4%) who received two or more treatments of RAI. Less than 10% of patients received other forms of radiotherapy (external beam radiation therapy, ablation therapy, or brachytherapy) and thyroid hormone replacement therapy with levothyroxine. RAI-R status was mainly confirmed by progression of metastatic disease despite significant concentrations of RAI (39.6%), or all or some lesions not able to concentrate RAI. At the time of data abstraction, 75.0% of patients were still alive, while 23.1% of patients had died and of those deaths 62.0% were attributed to RAI-R DTC.

### Lenvatinib treatment patterns

Overall, 62.0% of patients initiated lenvatinib treatment at the recommended starting dose of 24 mg daily [13], and the remaining patients initiated at 14 mg to 20 mg once daily doses. Most patients (90.3%) remained on their respective starting doses during their treatment. Approximately 8% of patients required a dose alteration (increase, decrease, or treatment interruption) during lenvatinib treatment. The median time of follow-up was 18.9 months (IQR: 12.3–24.7). By the end of follow-up, 1L treatment with lenvatinib was permanently discontinued in 32.5% of patients and was still ongoing in 67.5% of patients.

The median duration of lenvatinib therapy by descriptive analyses was 17.5 months (IQR: 8.8–25.0) overall, 9.1 months (IQR: 5.1–16.1) in patients who discontinued therapy, and 20.1 months (IQR: 15.9–27.8) in those still on therapy, although this latter value reflects the median duration of follow-up (18.9 months) more than a clear estimate of time to treatment discontinuation. Among the 100 patients who permanently discontinued 1L lenvatinib treatment, the main reasons for discontinuation were reported as disease progression (38.0%), death (33.0%), inadequate response or loss of response (14.0%), patient request (11%), and poor performance status (7%). Following discontinuation, 19 patients started a second line treatment with sorafenib, cabozantinib, pralsetinib, or selpercatinib.

### Clinical outcomes

**Fig. 1:**
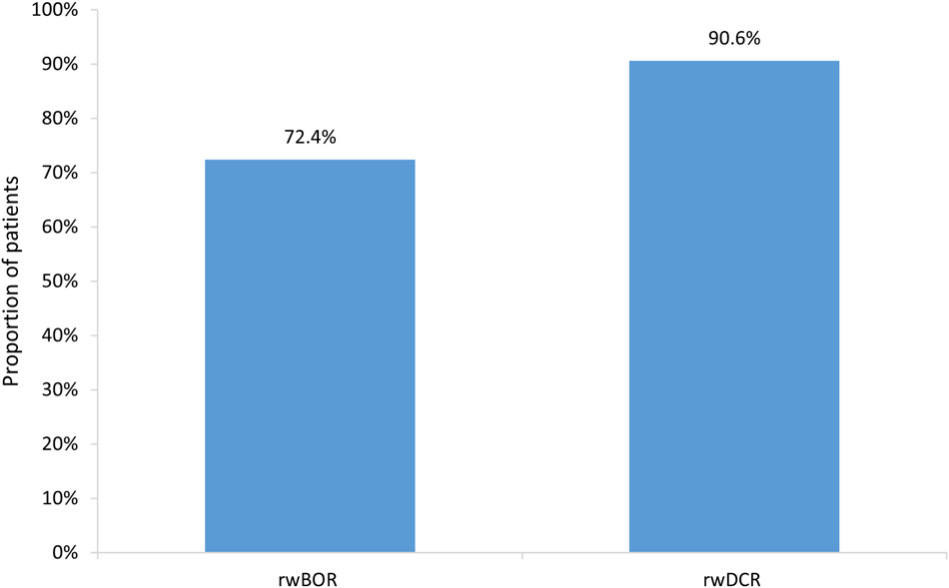
Real-world best overall response and disease control rate of 1L lenvatinib monotherapy-treated RAI-R DTC patients

Based on physician-reported response to lenvatinib treatment, rwORR was 72.4%, and rwDCR was 90.6% (Table 2; Fig. 1). Most physicians considered multiple factors when assessing treatment response to lenvatinib, including objective criteria (Response Criteria in Solid Tumors [RECIST] v1.0, RECIST v1.1, or iRECIST) [14–16], imaging (computed tomography [CT], positron emission tomography [PET], magnetic resonance imaging [MRI]), other patient and clinical factors (patient symptoms, physical performance status), or a combination of these criteria (Table 2).

**Table 2:** Assessment criteria and real-world treatment response in patients with RAI-R DTC following initiation of 1L lenvatinib

	Overall (*N* = 308)
Response evaluation criteria, *n* (%)	
RECIST v1.0	37 (12.4)
RECIST v1.1	171 (57.2)
iRECIST	22 (7.4)
Physician judgment based on radiology	6 (2.0)
Imaging, *n* (%)	
CT	143 (47.8)
PET	128 (42.8)
MRI	46 (15.4)
MRS	10 (3.3)
Ultrasound	9 (3.0)
Skeletal scintigraphy	1 (0.3)
Patient/clinical factors, *n* (%)	
Patient symptoms	145 (48.5)
Patient performance status	111 (37.1)
Basal thyroglobulin (Tg)	44 (14.7)
Tg antibodies (TgAb)	19 (6.4)
Recombinant human thyrotropin (rhTSH) stimulated Tg levels	16 (5.4)
Best response recorded, *n* (%)	
Complete response	83 (26.9)
Partial response	140 (45.5)
Objective response rate	223 (72.4)
Stable disease	56 (18.2)
Disease control rate	279 (90.6)
Disease progression	20 (6.5)

Note: these criteria are not mutually exclusive

*1L* first line, *CT* computed tomography, *MRI* magnetic resonance imaging, *MRS* magnetic resonance spectrometry, *PET* positron emission tomography, *RAI-R DTC* radioiodine-refractory differentiated thyroid cancer, *RECIST* Response Criteria in Solid Tumors

**Fig. 2:**
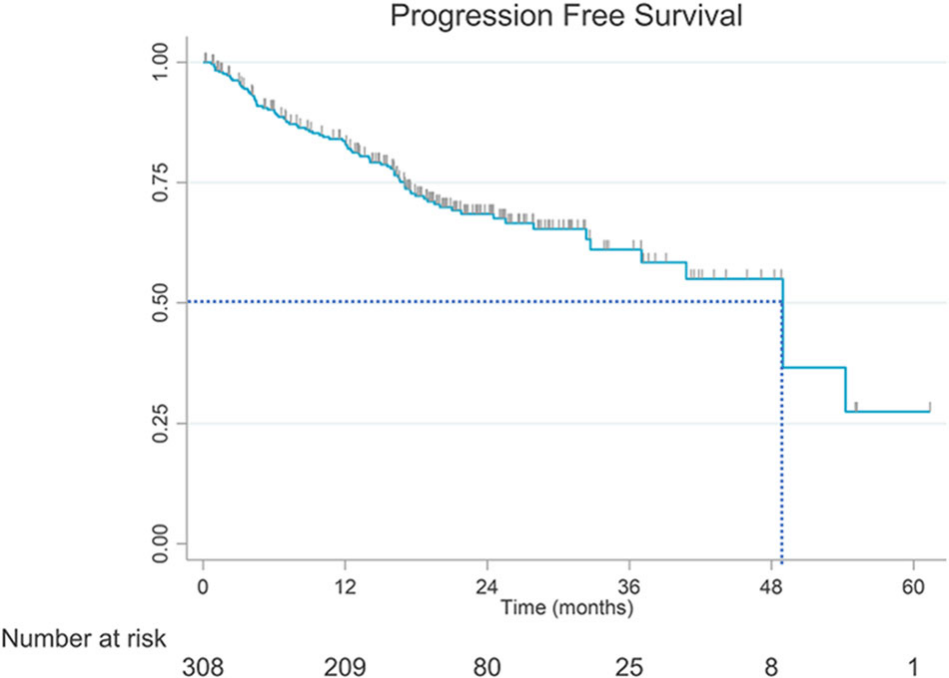
Real-world progression-free survival from initiation of 1L lenvatinib monotherapy in RAI-R DTC patients

**Fig. 3:**
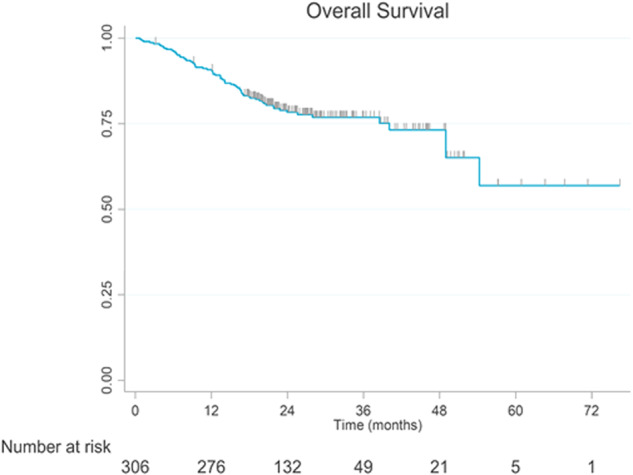
Overall survival from initiation of 1L lenvatinib monotherapy in RAI-R DTC patients

Median rwPFS (87 events, 221 censored) was 49.0 months (95% CI: 37.0-not estimable), with 83.3% (95% CI: 78.3–87.2) of patients estimated to be progression-free and alive at 12 months, 68.5% (95% CI: 62.0–74.1) at 24 months, 55.0% (95% CI: 43.2–65.3) at 48 months, and 27.5% (95% CI: 8.7–50.6) at 60 months (Fig. 2). By the end of follow-up, 75% of patients were alive, with estimated OS rates of 90.8% (95% CI: 87.0–93.6) at 12 months, 78.4% (95% CI: 73.0–82.8) at 24 months, 73.2% (95% CI: 65.5–79.5) at 48 months, and 57.0% (95% CI: 36.8–72.8) at 72 months. Median OS (69 events, 237 censored) was not reached (Fig. 3).

## Discussion

This retrospective real-world study reported treatment patterns and clinical outcomes of over 300 RAI-R DTC patients who received 1L lenvatinib monotherapy in US clinical practice. The study included a broad, representative patient population from all geographic regions in the US regardless of age, race, and physical performance status. The study period was between 2015–2020 (for eligible patients to initiate lenvatinib treatment), allowing each patient to have at least one year and as long as seven years of follow-up since the initiation of lenvatinib (if alive and not lost to follow-up). Thus, we believe our evaluation of treatment patterns and clinical outcomes can be considered accurate.

While some practitioners may prefer to start lenvatinib at lower dosages due to concern for tolerance, published data support starting at the approved dosage. In a two-arm study comparing the safety and efficacy of a lower 18 mg once daily dose of lenvatinib in RAI-R DTC patients with lenvatinib 24 mg once daily, the ORR in the 24 mg once daily arm was 57.3% and 40.3% in the 18 mg once daily arm, with a similar frequency of adverse events [17]. In our real-world study, approximately two thirds of the patients initiated 1L lenvatinib at a 24 mg once daily dose, which would explain why responses were so high, with 90% of all patients remaining on their individual starting dosage regimen.

We also found that the median duration of lenvatinib treatment was 17.5 months, which was numerically higher than the median duration of lenvatinib treatment in the SELECT trial (13.8 months) [9], possibly due to potential differences between the included patient populations, with 75% of our patients still receiving 1L lenvatinib by the end of follow-up. Moreover, while the assumption is made that patients that were selected for treatment had symptomatic progression, although this was not a requirement. Additionally, overall tumor burden could not be assessed. Together, the population in our study may have been generally healthy, allowing for greater tolerability of higher doses and prolongation of treatment with lenvatinib.

Most patients achieved benefit from treatment with lenvatinib, as 72% attained a CR or PR. This was comparable to the 65% seen in the lenvatinib arm of the SELECT trial [9]. It is worth noting that, in our study, treatment response to lenvatinib was collected from patient’s medical record as reported by the managing physician. It is acknowledged that evaluation based on RECIST criteria, whilst commonly utilized in clinical trials for evaluating treatment effect [12, 14], may not be strictly followed for disease evaluation in clinical practice.

As observed in our study, there were differences in the assessment criteria used among participating oncologists in real world clinical practice, with each decision being personalized, multi-factor consideration. In addition, as listed in RECIST v1.1, confirmation of CR and PR may be required in some trials, making the evaluation criteria stricter in such settings. The evaluation timing and frequency depend on patient’s office visit, which may be different in clinical practice vs. clinical trials, leading to potential differences in the reported treatment response.

The rwORR of 72% in our patient cohort was also similar to that reported in a real-world study of RAI-R DTC patients in the US by Kish et al., in which a 65% ORR was achieved in those remaining on lenvatinib versus 54% in those patients who discontinued lenvatinib [18]. In both studies, disease response data were verified by radiographic imaging and tumor lesion measurements in accordance with RECIST v1.1 guidelines [14].

Supportive care of patients with DTC in the US has improved over the last few decades [3], and it is to be expected that the patient prognosis may be more favorable than in early clinical trials due to improved management and expectation of side effects. Moreover, as TKI therapies become more commonplace in the treatment of many oncologic diseases, clinicians have gained more confidence in managing side effects, permitting patients to remain on drug for longer periods; this may explain why the rwDCR in our study was 90.6%. Similarly, the median rwPFS was estimated to be 49 months, and more than half of the patients were expected to be alive past 6 years. Previous real-world studies of lenvatinib conducted in the US and other countries reported median PFS ranging from 10 to 35 months. With these experiences, however, there could be differences in the patient populations as some of these studies included patients who were treated with prior lines of multi-kinase inhibitor therapies [19–21].

Our study has several strengths: the inclusion of a large patient sample from all practice types (community, academic) across all geographic regions of the US resulted in a diverse patient population, similar to the sample size in the pivotal phase 3 trial [9]. Almost 32% of our study participants were from underserved populations, which far exceeds the rates of enrollments into oncology studies in the US. Recently published data from 2017 to 2022 demonstrate that only 4.4% and 4.2% of the Black and Latino patients participated in oncology trials, respectively [22]. Finally, the design of our real-world study also allowed for sufficient follow-up data that were critical for assessing survival outcomes.

### Limitations

Several potential limitations should be considered when assessing the study findings. Firstly, our study is subject to potential physician selection bias as only oncologists who met the study eligibility criteria and consented to participate provided patient data for this study. Efforts were made to minimize the bias by recruiting a physician sample from across all regions in the US and allowing a maximum of 3 oncologists per practice, so patient data could be collected from a broad physician sample to minimize the impact of a few practices. Secondly, our study is subject to potential patient selection bias as each physician provided data for approximately five patients, which may not represent all potentially eligible patients, with physicians in urban areas likely to see more patients overall. Efforts were made to minimize the bias by instructing physicians to randomly select from all eligible patients. Thirdly, it is expected that there could be differences in clinical outcome assessment schedules and criteria used among participating oncologists in real-world clinical practice, especially for the assessment of treatment response and progression. Finally, adverse events from retrospective study designs do not normally need to be reported as individual case safety reports, since it is not possible to make a causality assessment. For this reason, we were not able to report any events that could be attributed to lenvatinib.

## Conclusion

The results from our real-world retrospective patient chart review provide evidence on treatment characteristics and related clinical outcomes among patients with RAI-R DTC treated with 1L lenvatinib monotherapy between 2015–2020 in clinical practice in the US. Our study reinforces the clinical effectiveness of 1L lenvatinib monotherapy as a standard of care in patients with RAI-R DTC in real-world clinical practice.
